# Air Pollutant and Health-Efficiency Evaluation Based on a Dynamic Network Data Envelopment Analysis

**DOI:** 10.3390/ijerph15092046

**Published:** 2018-09-18

**Authors:** Tao Zhang, Yung-ho Chiu, Ying Li, Tai-Yu Lin

**Affiliations:** 1 West China School of Public Health, Sichuan University, Wangjiang Road No. 29, Chengdu 610064, China; scdxzhangtao@163.com; 2 Department of Economics, Soochow University, 56, Kueiyang St., Sec. 1, Taipei 100, Taiwan; echiu@scu.edu.tw; 3 Business School, Sichuan University, Wangjiang Road No. 29, Chengdu 610064, China; 4 Department of Economics, Soochow University, 56, Kueiyang St., Sec. 1, Taipei 100, Taiwan; eickyla@gmail.com

**Keywords:** air pollutant emissions, Dynamic Network SBM (DNSBM) Model, efficiency, health expenditure

## Abstract

Environmental pollution and the associated societal health issues have attracted recent research attention. While most research has focused on the effect of air pollution on human health and local economies, few articles have discussed the environment, health, and economic development in in an integrated analysis. This paper used a Dynamic Network SBM Model to evaluate production and health efficiencies in Chinese cities and found that the production efficiency scores were slightly higher than the health efficiency scores, with the two-stage efficiency scores in most cities having significant fluctuations. Labor, fixed assets, energy, GDP, and lung disease and mortality reduction efficiencies in the first stage were generally high; however, the medical input efficiencies in the second stage were low, indicating that there was there significant room for improvement in many cities.

## 1. Introduction

Air pollution is one the most worrying environmental issues in developing countries. Since 2013, the atmospheric pollution; carbon dioxide, sulfur dioxide, and nitrogen oxides; resulting from rapid industrial development has damaged the health of local communities. China’s rapid economic development over the past few decades has resulted in serious air pollution and greenhouse gas emissions. Table 1 shows the emissions from six major air pollutants; SO_2_, NO_2_, PM_10_, PM_2.5_, O_3_ and CO; in mainland China (not including Hong Kong, Macau and Taiwan) from 2013, when the newest air quality standards were put into practice, to 2017 [1]. As can be seen almost all air pollutants except for O_3_ fell, with the reduction in SO_2_ being the highest (55.5%). These reductions are the result of multiple government interventions to combat air pollution; the three-year plan for defending the blue sky, the promotion of green development, and enhanced environmental law enforcement. However, air pollution remains a serious problem in China as even after these significant interventions, the PM_10_ and PM_2.5_ concentration levels have not yet reached the national secondary standard, therefore, more concerted efforts are needed.

Data collected by the World Meteorological Organization (WMO) Global Atmosphere Monitoring System show that the CO_2_, methane, and nitrous oxide concentrations today are 145%, 257%, and 122% higher than before industrialization [2]. Excessive carbon dioxide emissions have been a major contributor to climate change, which has resulted in polar ice reductions, sea level rises, and soil salinization in coastal areas. The temperature increases have also resulted in decreased mountain snow cover and reduced water resources. After signing the Paris Agreement, many countries have implemented policies to reduce emissions in an effort to reduce the climate change effects.

Past research on air pollution has focused on association assessments [3,4], risk assessments [5,6], health impact assessments [7], life cycle assessments [8,9], or the effect of air pollution on public health and the social economy in terms of the relationship between environmental pollution, diseases, and social economics. However, there has been little research that has examined the coordinated effect of the environment, health, and economic development from an efficiency perspective. To address this research gap, this paper used a Dynamic Network SBM (DNSBM) Model to examine data from 2013 to 2016 in 31 Chinese cities and assess the combined environmental and health efficiencies. In the first stage (production stage), labor, fixed assets, and energy consumption are the input indicators and GDP is the output indicator, with the links to the health stage being CO_2_ and AQI variables. In the second stage (health stage), health expenditure is the input and birth rate is the output variable, with the carry-over variables being the respiratory diseases and the mortality rate reductions.

The remainder of this paper is organized as the follows: Section 2 gives a comprehensive literature review, Section 3 describes the research method, Section 4 gives the comprehensive empirical results and discussion, and Section 5 gives the conclusions and policy proposals.

## 2. Literature Review

### 2.1. Air Pollution Intervention, Public Health and the Social Economy

Air pollution has been identified as a significant risk factor for various respiratory and cardiovascular diseases. A meta-analysis [10] found that outdoor air pollution was a major contributor to the increase in chronic obstructive pulmonary diseases (COPD). Clinical evidence from China, the United States and the European Union concluded that a 10 µg/m^3^ increase in PM_10_ led to a significant rise in COPD exacerbation and deaths. In another study, [11] epidemiological and pathobiological evidence confirmed that both short and long-term exposure to air pollution increased stroke risk, and in other studies, it was found that air pollution could also be associated with other diseases and symptoms, such as still births [12], depression [13] and Alzheimer’s [14].

Given the serious air pollution impacts on public health, a rise in pro-environment attitudes in society has led governments to consider a willingness-to-pay (WTP) to improve air quality [15]. Four main strategies have been suggested to improve air quality; general air quality control regulations, traffic related emissions controls, energy related emissions controls, and greenhouse gas emissions controls [16]. However, as air quality interventions can result in social and economic problems such as inconvenient traffic, factory bankruptcies, and unemployment [17], governments need to weigh up the costs and benefits associated with air pollution interventions so as to balance air pollution controls and socio-economic development.

Generally, joint assessments of air pollution interventions, public health, and the socio-economic effects need to include four assessment types; association assessments, risk assessments, health impact assessments, and efficiency assessments.

#### 2.1.1. Association Assessments

An association assessment uses mathematical modeling and statistical analysis to characterize the distributional associations between air pollution, public health and social economy. Luo et al. [3] analyzed the relationship between economic conditions and air pollutants in large Chinese cities and found that there was a U-shaped curve for the relationship in which the environmental quality first declined and then improved with income growth. Greenfield et al. [4] found that that while the environmental and socioeconomic factors were both significantly associated with the disease burden, the socio-economic factors were more closely associated with the disease burden than the environmental factors.

A common misuse of association assessments can been to make causal inferences based solely on the associations. Technically, association assessments involve an examination of the statistical distributions of multiple factors; however, causal inferences focus on the treatment effects of certain interventions [18]. This difference between association and causation can be exemplified by differences between sunglasses and sunstroke; that is sunglasses can be *associated* with sunstroke, but do not cause sunstroke. As air pollution control decision making requires a proof of causation rather than association, it is vital to keep these differences in mind.

#### 2.1.2. Risk Assessment

Risk assessment is a type of causal inference; for example for air pollution, it involves an identification of the air pollution risks, the potential consequences, the occurrence probabilities, the tolerability or acceptability of the air pollution risks, and methods for mitigating or reducing air pollution risk probabilities.

One of the most widely used risk assessment approaches has been a comparative risk assessment framework that estimates the levels and trends in exposure, deaths, and disability-adjusted life-years (DALYs) from risk factors that include but are not limited to air pollution. Smith et al. gave details about the methods used in the comparative risk assessment of household air pollution, such as how the exposure-response model was built to estimate the relative risks.

However, risk assessment models for outdoor air pollution are far less well developed [5,6], primarily because outdoor air pollution exposure is much more difficult to measure. Dreaves claimed that there was less focus on this type of risk assessment because of poor stakeholder involvement and participation [7].

#### 2.1.3. Health Impact Assessment

Health impact assessments (HIA) are a combination of procedures, methods and tools to assess the potential effects of a policy, program, or project on the health of a population and the distribution of those effects within the population [7].

There have been many methodological HIA iterations, and while there has not been a single agreed HIA method, it is generally accepted that an HIA should include [19] screening (establishing whether an HIA is required), scoping (planning what to do and how to do it), appraisal (identifying the health hazards and considering impact evidence), reporting (developing recommendations to reduce hazards and/or improve health), and monitoring (monitoring the implementation of the proposal and evaluating whether the HIA has influenced the decision making process). For instance, the Aphekom project used traditional HIA methods to assess the health impact of air pollution in 25 European cities, finding that life expectancy and monetary benefits increased significantly beyond the achievements of current EU legislation when fine particle levels were reduced in European cities and pollution was regulated near busy roads.

HIAs can also have drawbacks. As HIAs emphasize the outcomes (*impact*) of a decision process, when confronted with complicated real-world cases, they lack adequate methods to measure and quantify the inputs, making it difficult to holistically evaluate the efficiency of each proposal. Birley, for example, criticized HIAs for failing to validate scientific hypotheses and stop proposals (however inappropriate) [20].

#### 2.1.4. Life Cycle Assessment

As there are many interventions available to reduce the negative effects of air pollution on the environment and public health, it is necessary to select the most efficient. Life-cycle assessments (LCA) have been a key efficiency assessment method as these are able to evaluate the potential impacts associated with air pollution interventions and interpret the results to help make informed decisions. The LCA generally had four main phrases; goal and scope definitions, an inventory analysis, an impact assessment, and interpretation [21].

A recent study examined the environmental effects of PM_2.5_ on human health and identified this factor as an important for life cycle impact assessments [8]. The effect of PM on health in the U.S. in 2005 was estimated at 130,000 deaths and 2 million DALY. In another study, Damgaard et al. [9] compared eight different air pollution control technologies using lifecycle-assessment modelling and found that the potential environmental air emissions impacts had decreased over the last 35 years and that these impacts could be partly or fully offset by recovering the energy produced from fossil fuels.

However, as LCA methods are generally only able to assess a single exposure type or intervention, they are unable to comprehensively assess both economic and health efficacy at the same time. Further as LCA allows researchers to choose the parameters and data, there is a risk of conflict when seeking to compare assessments as a favored proposal in one study may be disfavored in another study because of different parameters and data.

### 2.2. Summary and Implications of the Literature Review

The literature review revealed that air pollution is an environmental, health, and socio-economic issue. Previous research has provided sound evidence for the relationship between air pollution, health, and the social economy and given examples of successful policies and regulations that have sought to reduce the negative effects. However, even though there are available references, experiences, and paradigms to assess air pollution control in China, it is necessary to determine which if any are the most appropriate at this time. In this paper, however, we have chosen to analyze effectiveness using efficiency analyses, as these have been insufficient in previous research. Therefore, to examine the association between air pollution, health, and the social economy, this study proposes a new method to holistically evaluate socio-economic and health efficiencies under the same framework.

## 3. Research Methods

As in Tone and Tsutsui [22], a two-stage Dynamic Network DEA model was designed for the input and output efficiency indicators. In this section, the Dynamic Network DEA model is analyzed first, after which the two stage input and output efficiency indicators are described and assessed.

### 3.1. Dynamic Network DEA

Data Envelopment Analysis is based on a Pareto optimal solution concept and uses linear programming techniques to evaluate the relative efficiencies of Decision Making Units (DMU). The first use of this type of research method was 1957 in Farrell’s [23] study on the “Measurement of Productive Efficiency”. However, Farrell’s efficiency assessment model was only suitable for a single input and a single output. Because such research generally involves multiple inputs and multiple outputs, Charnes, Cooper and Rhodes [24] proposed the CCR model in 1978, which extended Farrell’s model to allow for multiple inputs and multiple outputs, with the optimal solution being solved using linear programming, which is now known as Data Envelopment Analysis. In 1984, Banker, Charnes and Cooper [25] proposed the BCC model that included a variable return to scale (VRS) assumption to replace the constant return to scale (CRS) assumption. Tone [26] then proposed a Slacks-Based Measure (SBM) to allow for any output and input slacks. Using non-radial estimation and a single scalar to present the SBM efficiency, the efficiency values are typically between 0 and 1; when the efficiency of a decision making unit is 1, there is no input or output slack on the production frontier.

Färe et al. [27] proposed Network Data Envelopment Analysis (NDEA) in 2007, in which the production processes were broken up into secondary production technologies called Sub-DMUs, and traditional CCR or BCC models employed to determine the optimal solutions. In 2013, Tone and Tsutsui [22] proposed the SBM (weighted slack-based measures) Dynamic Network DEA, which was based on the links between the decision-making units, included each department as a Sub-DMU, and had carry-over link activities.

*Dynamic Network DEA Model and Solution:*

It is assumed that the number of DMUs is *n* (*j* = 1, ..., *n*), with each DMU being divided into a number of *k*, (*k* = 1, …, *K*), and time periods *t*, (*t* = 1, …, *T*). Each DMU has an input and output in period *t* through a carry over (link) to the next period *t* + 1.

Set *m_k_* and *r_k_* as the input and output for each division *K*, in which (*k,h*)*i* indicates division *k* to *h*, and *L_hk_* denotes the set of *k* and *h*.

*Inputs and Outputs*

$X_{ijk}^{t}\in R_{+}^{}$$\left(i=1,\ldots ,m_{k}^{};\;j=1,\ldots ,n;K=1...,K;t=1,\ldots ,T\right)$: indicates input i in period t for division k in $DMU_{j}$.

$y_{rjk}^{t}\in R_{+}^{}$$\left(r=1,\ldots ,r_{k}^{};\;j=1,\ldots ,n;K=1...,K;t=1,\ldots ,T\right)$: indicates output r in period t for division k in $DMU_{j}$.

If part of the output is not good, it is considered an input to division k.

*Links*

$Z_{j(kh)t}^{t}\in R_{+}^{}$$(j=1;\ldots ;n;l=1;..;L_{hk}^{};t=1;\ldots ;T)0$: denotes the link between division k and division h in $DMU_{j}$ in period t, where $L_{hk}^{}$ is the number of links between k and h.

*Z^t^_j_*_(*kh*)*t*_ ∊ *R*_+_ (*j* = 1; …; *n*; *l* = 1; …; *L_kh_*; *t* = 1; …; *T*).

*Carry-overs*

$Z_{jkl}^{\left(t,t+1\right)}\in R_{+}^{}$$\left(j=1,\ldots ,n;l=1,..,L_{k}^{};k=1,\ldots k,t=1,\ldots ,T-1\right)$: denotes the carry-overs from division k to h in $DMU_{j}$ from period t to t+1 where $L_{k}^{}$ is the number of carry-overs from division k.

#### Objective Function

*Overall Efficiency:*

$$\theta_{0}^{*}=\min\frac{\sum_{t=1}^{T}W^{t}\left[\sum_{k=1}^{K}W^{k}\left[1-\frac{1}{m_{k}+linkin_{k}+nbad_{k}}(\sum_{i=1}^{m_{k}}\frac{S_{iok}^{t-}}{x_{iok}^{t}}+\sum_{{\left(kh\right)}_{l}=1}^{linkin_{k}}\frac{s_{o{\left(kh\right)}_{l}in}^{t}}{z_{o{\left(kh\right)}_{l}in}^{t}}+\sum_{k_{l}=1}^{nbad_{k}}\frac{s_{ok_{l}bad}^{\left(t,(t+1)\right)}}{z_{ok_{l}bad}^{\left(t,(t+1)\right)}}\right]\right]}{\sum_{t=1}^{T}W^{t}\left[\sum_{k=1}^{K}W^{k}\left[1+\frac{1}{r_{k}+linkout_{k}+ngood_{k}}(\sum_{r=1}^{r_{k}}\frac{s_{rok}^{t+}}{y_{rok}^{t}}+\sum_{{\left(kh\right)}_{l}=1}^{linkout_{k}}\frac{s_{o{\left(kh\right)}_{l}out}^{t}}{z_{o{\left(kh\right)}_{l}out}^{t}}+\sum_{k_{l}=1}^{ngood_{k}}\frac{s_{ok\mathrm{lg}ood}^{\left(t,(t+1)\right)}}{z_{ok\mathrm{lg}ood}^{\left(t,(t+1)\right)}}\right]\right]}$$

Constraints:

$x_{ok}^{t}=X_{k}^{t}\lambda_{k}^{t}+s_{ko}^{t-}$ (∀k,∀t)

$y_{ok}^{t}=Y_{k}^{t}\lambda_{k}^{t}-s_{ko}^{t+}$ (∀k,∀t)

$e\lambda_{k}^{t}=1$ (∀k,∀t)

$\lambda_{k}^{t}\geq 0,$ $s_{ko}^{t-1}\geq 0,$ $s_{ko}^{t+}\geq 0,$ (∀k,∀t)

$Z_{(kh)free}^{t}\lambda_{h}^{t}=Z_{(kh)free}^{t}\lambda_{k}^{t}$ (∀(k,h)free,∀t)

$Z_{(kh)free}^{t}=(Z_{1(kh)free}^{t},\ldots ,Z_{n(kh)free}^{t})\in R^{L_{(h)free}\times n}$

$Z_{o(kh)fix}^{t}=Z_{(kh)fix}^{t}\lambda_{h}^{t}$ (∀(k,h)fix,∀t)

$Z_{o(kh)fix}^{t}=Z_{(kh)fix}^{t}\lambda_{k}^{t}$ (∀(k,h)fix,∀t)

$Z_{o(kh)in}^{t}=Z_{(kh)in}^{t}\lambda_{k}^{t}+S_{o(kh)in}^{t}$ $\left((kh)in=1,\ldots ,linkin_{k}\right)$

$Z_{o(kh)out}^{t}=Z_{(kh)out}^{t}\lambda_{k}^{t}-S_{o(kh)out}^{t}$ $\left((kh)out=1,\ldots ,linkout_{k}\right)$

$\sum_{j=1}^{n}z_{jk_{1}\alpha }^{\left(t,(t+1)\right)}\lambda_{jk}^{t}=\sum_{j=1}^{n}z_{jk_{1}\alpha }^{\left(t,(t+1)\right)}\lambda_{jk}^{t+1}$ $\left(\forall k;\forall k_{l};t=1,\ldots ,T-1\right)$

$Z_{ok_{l}good}^{\left(t,(t+1)\right)}={\sum_{j=1}^{n}z}_{jk_{l}good}^{\left(t,(t+1)\right)}\lambda_{jk}^{t}-s_{ok_{l}good}^{\left(t,(t+1)\right)}$ $k_{l}=1,\ldots ,ngood_{k};\forall k;\forall t)$

$Z_{ok_{l}bad}^{\left(t,(t+1)\right)}={\sum_{j=1}^{n}z}_{jk_{l}bad}^{\left(t,(t+1)\right)}\lambda_{jk}^{t}-s_{ok_{l}bad}^{\left(t,(t+1)\right)}$ $k_{l}=1,\ldots ,nbad_{k};\forall k;\forall t)$

$Z_{ok_{l}free}^{\left(t,(t+1)\right)}={\sum_{j=1}^{n}z}_{jk_{l}free}^{\left(t,(t+1)\right)}\lambda_{jk}^{t}-s_{ok_{l}free}^{\left(t,(t+1)\right)}$ $k_{l}=1,\ldots ,nfree_{k};\forall k;\forall t)$

$Z_{ok_{l}fix}^{\left(t,(t+1)\right)}={\sum_{j=1}^{n}z}_{jk_{l}fix}^{\left(t,(t+1)\right)}\lambda_{jk}^{t}-s_{ok_{l}fix}^{\left(t,(t+1)\right)}$ $k_{l}=1,\ldots ,nfix_{k};\forall k;\forall t)$

(1) $$s_{ok_{l}good}^{\left(t,(t+1)\right)}\geq 0,s_{ok_{l}bad}^{\left(t,(t+1)\right)}\geq 0,s_{ok_{l}free}^{\left(t,(t+1)\right)}:free(\forall k_{l};\forall t)$$

*Period and Division Efficiencies*

*Period efficiency:*

$$\tau_{0}^{t^{*}}=\min\frac{\sum_{k=1}^{K}W^{k}\left[1-\frac{1}{m_{k}+linkin_{k}+nbad_{k}}(\sum_{i=1}^{m_{k}}\frac{S_{iok}^{t-}}{x_{iok}^{t}}+\sum_{{\left(kh\right)}_{l}=1}^{linkin_{k}}\frac{s_{o{\left(kh\right)}_{l}in}^{t}}{z_{o{\left(kh\right)}_{l}in}^{t}}+\sum_{k_{l}=1}^{nbad_{k}}\frac{s_{ok_{l}bad}^{\left(t,(t+1)\right)}}{z_{ok_{l}bad}^{\left(t,(t+1)\right)}}\right]}{\sum_{k=1}^{K}W^{k}\left[1+\frac{1}{r_{k}+linkout_{k}+ngood_{k}}(\sum_{r=1}^{r_{k}}\frac{s_{rok}^{t+}}{y_{rok}^{t}}+\sum_{{\left(kh\right)}_{l}=1}^{linkout_{k}}\frac{s_{o{\left(kh\right)}_{l}out}^{t}}{z_{o{\left(kh\right)}_{l}out}^{t}}+\sum_{k_{l}=1}^{ngood_{k}}\frac{s_{ok_{l}good}^{\left(t,(t+1)\right)}}{z_{ok_{l}good}^{\left(t,(t+1)\right)}}\right]}$$

*Division efficiency:*

$$\delta_{0t}^{*}=\min\frac{\sum_{t=1}^{T}W^{t}\left[1-\frac{1}{m_{k}+linkin_{k}+nbad_{k}}(\sum_{i=1}^{m_{k}}\frac{S_{iok}^{t-}}{x_{iok}^{t}}+\sum_{{\left(kh\right)}_{l}=1}^{linkin_{k}}\frac{s_{o{\left(kh\right)}_{l}in}^{t}}{z_{o{\left(kh\right)}_{l}in}^{t}}+\sum_{k_{l}=1}^{nbad_{k}}\frac{s_{ok_{l}bad}^{\left(t,(t+1)\right)}}{z_{ok_{l}bad}^{\left(t,(t+1)\right)}}\right]}{\sum_{t=1}^{T}W^{t}\left[1+\frac{1}{r_{k}+linkout_{k}+ngood_{k}}(\sum_{r=1}^{r_{k}}\frac{s_{rok}^{t+}}{y_{rok}^{t}}+\sum_{{\left(kh\right)}_{l}=1}^{linkout_{k}}\frac{s_{o{\left(kh\right)}_{l}out}^{t}}{z_{o{\left(kh\right)}_{l}out}^{t}}+\sum_{k_{l}=1}^{ngood_{k}}\frac{s_{ok_{l}good}^{\left(t,(t+1)\right)}}{z_{ok_{l}good}^{\left(t,(t+1)\right)}}\right]}$$

*Division Period efficiency:*

$$p_{0k}^{t*}=\frac{1-\frac{1}{m_{k}+linkin_{k}+nbad_{k}}(\sum_{i=1}^{m_{k}}\frac{S_{iok}^{t-}}{x_{iok}^{t}}+\sum_{{\left(kh\right)}_{l}=1}^{linkin_{k}}\frac{s_{o{\left(kh\right)}_{l}in}^{t}}{z_{o{\left(kh\right)}_{l}in}^{t}}+\sum_{k_{l}=1}^{nbad_{k}}\frac{s_{ok_{l}bad}^{\left(t,(t+1)\right)}}{z_{ok_{l}bad}^{\left(t,(t+1)\right)}}}{1+\frac{1}{r_{k}+linkout_{k}+ngood_{k}}(\sum_{r=1}^{r_{k}}\frac{s_{rok}^{t+}}{y_{rok}^{t}}+\sum_{{\left(kh\right)}_{l}=1}^{linkout_{k}}\frac{s_{o{\left(kh\right)}_{l}out}^{t}}{z_{o{\left(kh\right)}_{l}out}^{t}}+\sum_{k_{l}=1}^{ngood_{k}}\frac{s_{ok_{l}good}^{\left(t,(t+1)\right)}}{z_{ok_{l}good}^{\left(t,(t+1)\right)}}}\left(\forall k;\forall t\right)$$

(2) $$Z_{ol_{k}}^{\left(0,1\right)}=\sum_{j=1}^{n}Z_{jlk}^{\left(0,1\right)}\lambda_{jk}^{l}\left(\forall l_{k}\right)$$

From the above results, the overall efficiency, period efficiency, division efficiency and division period efficiency can be determined.

### 3.2. Fixed Assets, Labor, Energy Consumption, GDP, Health Expenditure, Birth Rate, Respiratory Disease, and Death Rate Efficiencies

Hu and Wang’s [28] total-factor energy efficiency index is employed here to overcome any possible biases in the traditional energy efficiency indicators. There are nine key features used in this present study: fixed assets, labor, energy consumption, GDP, health expenditure, birth rate, respiratory diseases and death rate. In our study, “I” represents area and “t” represents time.

#### 3.2.1. Fixed Asset Efficiency

Fixed asset efficiency is the ratio of target fixed asset input to actual fixed asset input, the model for which is:

$$\mathrm{Fixed}\;\mathrm{asset}\;\mathrm{efficiency}=\frac{\mathrm{Target}\;\mathrm{Fixed}\;\mathrm{asset}\;\mathrm{input}\;\left(i,\;t\right)}{\mathrm{Actual}\;\mathrm{Fixed}\;\mathrm{asset}\;\mathrm{input}\;\left(i,\;t\right)}.$$

If the target fixed asset input is equal to the actual input level, then the fixed asset efficiency equals 1, indicating efficiency; however, if the target fixed asset input is less than the actual input level, then the fixed asset efficiency is less than 1, indicating inefficiency.

#### 3.2.2. Labor Efficiency

Labor efficiency is the ratio of target labor input to actual labor input, the model for which is:

$$\mathrm{Labor}\;\mathrm{efficiency}=\frac{\mathrm{Target}\;\mathrm{Labor}\;\mathrm{input}\;\left(i,\;t\right)}{\mathrm{Actual}\;\mathrm{Labor}\;\mathrm{input}\;\left(i,\;t\right)}$$

If the target labor input is equal to the actual input level, then labor efficiency equals 1, indicating efficiency; however, if the target labor input is less than the actual input, then the labor efficiency is less than 1, indicating inefficiency.

#### 3.2.3. Energy Consumption Efficiency

Energy consumption efficiency is the ratio of target energy input to actual energy input, the model for which is:

$$\mathrm{Energy}\;\mathrm{consumption}\;\mathrm{efficiency}=\frac{\mathrm{Target}\;\mathrm{energy}\;\mathrm{input}\;\left(i,\;t\right)}{\mathrm{Actual}\;\mathrm{energy}\;\mathrm{input}\;\left(i,\;t\right)}$$

If the target energy input is equal to the actual input level, then energy consumption efficiency equals 1, indicating efficiency; however, if the target energy input is less than the actual input level, then the energy consumption efficiency is less than 1, indicating inefficiency.

#### 3.2.4. GDP Efficiency

GDP efficiency is the ratio of actual desirable GDP output to target desirable GDP output, the model for which is:

$$\mathrm{GDP}\;\mathrm{efficiency}=\frac{\mathrm{Actual}\;\mathrm{desirable}\;\mathrm{GDP}\;\mathrm{output}\;\left(i,\;t\right)}{\mathrm{Target}\;\mathrm{desirable}\;\mathrm{GDP}\;\mathrm{output}\;\left(i,\;t\right)}$$

If the target desirable GDP output is equal to the actual desirable GDP output level, then the GDP efficiency equals 1, indicating efficiency. If the actual desirable GDP output is less than the target desirable GDP output level, then the GDP efficiency is less than 1, indicating inefficiency.

#### 3.2.5. Health Expenditure Efficiency

Health expenditure efficiency is the ratio of target health expenditure input to actual health expenditure input, the model for which is:

$$\mathrm{Health}\;\mathrm{Expenditure}\;\mathrm{efficiency}=\frac{\mathrm{Target}\;\mathrm{Health}\;\mathrm{Expenditure}\;\mathrm{input}\;\left(i,\;t\right)}{\mathrm{Actual}\;\mathrm{Health}\;\mathrm{Expenditure}\;\mathrm{input}\;\left(i,\;t\right)}$$

If the target health expenditure input is equal to the actual health expenditure input level, then the health expenditure efficiency equals 1, indicating efficiency; however, if the target health expenditure input is less than the actual health expenditure input level, then the health expenditure efficiency is less than 1, indicating inefficiency.

#### 3.2.6. Birth Rate Efficiency

Birth rate efficiency is the ratio of actual desirable birth rate output to target desirable birth rate output, the model for which is:

$$\mathrm{Birth}\;\mathrm{Rate}\;\mathrm{efficiency}=\frac{\mathrm{Actual}\;\mathrm{desirable}\;\mathrm{Birth}\;\mathrm{Rate}\;\mathrm{output}\;\left(i,\;t\right)}{\mathrm{Target}\;\mathrm{desirable}\;\mathrm{Birth}\;\mathrm{Rate}\;\mathrm{output}\;\left(i,\;t\right)}$$

If the target desirable birth rate output is equal to the actual desirable birth rate output level, then the birth rate efficiency equals 1, indicating efficiency; however, if the actual desirable birth rate output is less than the target desirable birth rate output level, then the birth rate efficiency is less than 1, indicating inefficiency.

#### 3.2.7. Respiratory Disease Efficiency

Respiratory disease efficiency is the ratio of target undesirable respiratory disease output to actual undesirable respiratory disease output, the model for which is;

Respiratory

$$\mathrm{Diseases}\;\mathrm{efficiency}=\frac{\mathrm{Target}\;\mathrm{Respiratory}\;\mathrm{Disease}\;\mathrm{Undesirable}\;\mathrm{output}\;\left(i,\;t\right)}{\mathrm{Actual}\;\mathrm{Respiratory}\;\mathrm{Disease}\;\mathrm{Undesirable}\;\mathrm{output}\;\left(i,\;t\right)}$$

If the target undesirable respiratory disease output is equal to the actual undesirable respiratory disease output, then the respiratory diseases efficiency equals 1, indicating efficiency; however, if the target undesirable respiratory disease output is less than the actual undesirable respiratory disease output, then the respiratory disease efficiency is less than 1, indicating inefficiency.

#### 3.2.8. Death Rate Efficiency

The death rate efficiency is the ratio of target undesirable death rate output to actual undesirable death rate output, the model for which is;

$$\mathrm{Death}\;\mathrm{Rate}\;\mathrm{efficiency}=\frac{\mathrm{Target}\;\mathrm{Death}\;\mathrm{Rate}\;\mathrm{Undesirable}\;\mathrm{output}\;\left(i,\;t\right)}{\mathrm{Actual}\;\mathrm{Death}\;\mathrm{Rate}\;\mathrm{Undesirable}\;\mathrm{output}\;\left(i,\;t\right)}$$

If the target undesirable death rate output is equal to the actual undesirable death rate output, then the death rate efficiency equals 1, indicating efficiency; however, if the undesirable target death rate output is less than the actual undesirable death rate output, then the death rate efficiency is less than 1, indicating inefficiency.

## 4. Results and Discussion

### 4.1. Data Sources and Description

This study used panel data from 31 of the most developed cities in eastern and western China. The economic and social development data from 2013 to 2016 were collected from the Statistical Yearbook of China, the Demographics and Employment Statistical Yearbook of China, and the City Statistical Yearbooks. Air pollutant data were collected from the China Environmental and Protection Bureau Annual Reports and the China Environmental Statistical Yearbook. As the 31 sample cities varied widely in terms of population, industries, natural resources, meteorological conditions, and geographical positions, they were considered to be representative of the general air pollution emissions and treatment situations in China.

**Figure 1:**
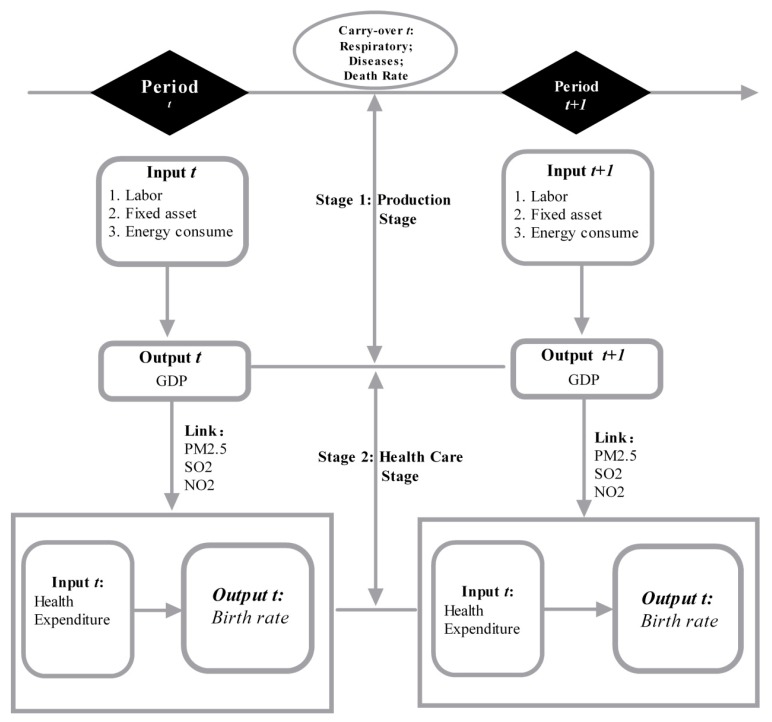
Network Dynamic Model.

Figure 1 shows the framework for the inter-temporal efficiency measurements and variables for the Network Dynamic Model.

The specific variables are explained in the following:

*Input variables:*

*Labor input (lab)*: numbers of employees in each city at the end of each year; unit = persons.

*Fixed Assets (assets)*: the capital stock in each city based on the fixed asset investment in each city; unit = 100 million CNY.

*Energy consumption (com)*: the total energy consumption in each city; unit = 100 million Dun.

*Output variable:*

*Desirable output (GDP)*: the GDP in each city; unit = 100 million CNY.

*Link Production Stage and health stage variables:*

*PM_2.5_*: atmospheric particulate matter (PM) with a diameter of less than 2.5 micrometers; unit = micrograms/cubic meter.

*SO_2_*: sulfur dioxide; released naturally by volcanic activity and produced as a by-product of the burning of fossil fuels contaminated with sulfur compounds.

*NO_2_;* Nitrogen dioxide (NO_2_); one of a group of highly reactive gases known as oxides of nitrogen or nitrogen oxides (N_X_). NO_2_, is an intermediate emission in the industrial synthesis of nitric acid, millions of tonnes of which are produced each year.

*Second stage health stage*

*Input variables:*

Government Health Expenditure

*Output variables:*

Birth rate

*Carry-over variables:*

Respiratory Disease and Death Rate

### 4.2. Input-Output Index Statistical Analyses

**Figure 2:**
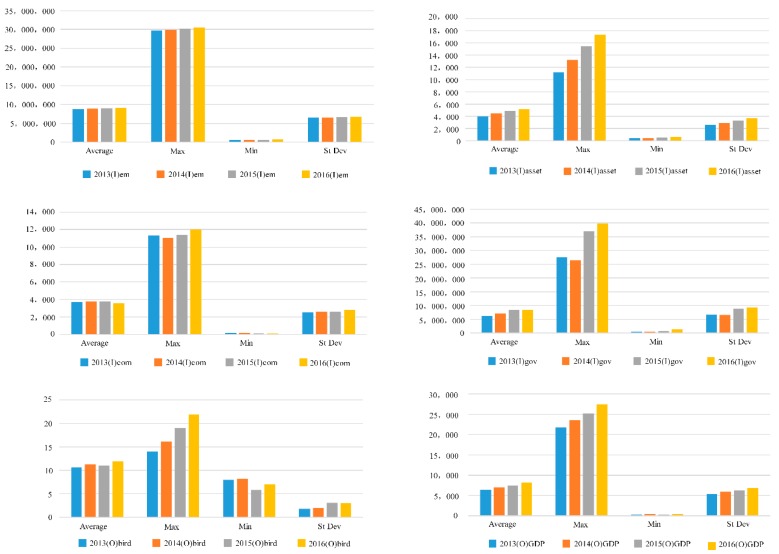
Input and output data from 2013–2016.

Figure 2 shows the statistical analysis of the input-output indicators. From 2013 to 2016, the growth in employment (em) was relatively slow, so the four years trend was slightly upward. The fixed assets input was large and significantly increased over the years. The maximum employed population increased slightly and the average and minimum employed population fluctuated.

While there were some fluctuations in energy consumption, the average growth was not significant and decreased in 2016, with the minimum decreasing significantly after 2014; however, maximum energy consumption had an upward trend, particular in 2016 and 2017. The gap between the maximum and minimum energy consumption was therefore increasing.

The government health expenditure input indicators also fluctuated, with the average increasing from 2013 to 2015 and falling in 2016. The maximum government Health Expenditure input in 2014 was slightly lower than in 2013, but increased significantly in 2015 and continued to increase in 2016. The birth rate maximum had a continuous upward trend with a significant overall increase; however, while the mean birth rate had a fluctuating upward trend, the minimum birth rate had a downward trend; therefore, the gap between the maximum and minimum birth rate was increasing.

The total GDP rose significantly over the years. While there was a significant increase in the maximum GDP, the average GDP was relatively flat, and the minimum GDP tended to fluctuate.

### 4.3. Total City Efficiency Scores for Each Year

Table 2 shows the total efficiency scores and ranks for 31 Chinese cities from 2013 to 2016. Five cities had total efficiency scores of 1; Beijing, Fuzhou, Guangzhou, Lhasa, Urumqi, and Yinchuan; Changsha, Nanning, and Wuhan had total efficiency scores around 0.95, and the total efficiency of the other cities was below 0.7. The lowest five ranked cities were Shijiazhuang, Haikou, Harbin, Chengdu, and Tianjin, all of which had total efficiency scores below 0.4.

Only three cities had continually rising efficiency; Guiyang, Kunming, and Nanjing; with all other cities having large efficiency fluctuations. Changchun’s efficiency rose significantly in 2016 from around 0.5 in 2015 to 0.88 in 2016. In 2016, Hefei’s efficiency rose to 0.89 from 0.65 in 2015 (the highest in the previous three years). Jinan’s efficiency was 0.5 or below in the first three years, but rose to 1 in 2016.

Several cities saw a decrease in their overall efficiency. Lanzhou’s total efficiency decreased from 0.89 in 2014 to 0.5 in 2015, the lowest level, before rising again to around 0.6 in 2016. Shanghai’s efficiency was 1 in 2013, but after 2014, fell to 0.63.

Some cities saw little or no change in their efficiency. Xining’s efficiency was around 0.7 in 2015 but below 0.5 in the other years. Tianjin’s efficiency was around 0.5 in 2013, rose to 0.65 in 2014, the highest, and then dropped again to around 0.40 in 2015 and 2016. Therefore, there were only 5 cities with an overall efficiency of 1, and all other 26 cities needed improvements, especially Shijiazhuang, Haikou, Harbin, Chengdu, and Tianjin.

### 4.4. Annual Efficiency Analysis at Each Stage

#### 4.4.1. Comparison of Total Efficiency, Stage Efficiency, Overall Rank, and Stage Rank

Table 3 shows the total efficiency scores and total ranks, the average efficiency scores, and the ranks in the two stages. Cities with efficiency below 0.5 in the first stage; Guiyang, Kunming, Shijiazhuang, Taiyuan, Xi’an, and Xining; were classified into one category, and cities with efficiency at 0.5 or greater were classified into another category. The cities with efficiencies greater than or equal to 0.5 in the first stage were embedded into the second stage; cities with efficiencies below 0.5 in the second stage were classified as “a city with an efficiency greater than 0.5 in the first stage but an efficiency less than 0.5 in the second stage” (In Table 3, these cities were Chengdu, Chongqing, Harbin, Haikou and Tianjin). “There were 20 (64.52% of our observations) cities with efficiencies greater than 0.5 in both stages”. After classifying the 31 provincial capital cities based on the above, the results were as follows:

(1) Characteristics of cities with efficiencies less than 0.5 in the first stage

From Table 3, there were six cities with efficiencies below 0.5 in the first stage; Guiyang, Kunming, Shijiazhuang, Taiyuan, Xi’an, and Xining. The main features of these cities were single industrial structures, a lack of characteristic industries, and with the main industries being labor intensive with high energy consumption. Therefore, these cities lack innovation and competitiveness, need to adjust their industrial structures away from traditional industries, and need to introduce new energy sources.

(2) Characteristics of cities with efficiencies greater than 0.5 in the first stage but below 0.5 in the second stage.

From Table 3, there were five cities in this category; Chengdu, Chongqing, Harbin, Haikou and Tianjin. The main characteristics were serious air pollution (Chengdu, Chongqing, and Tianjin), cities with large immigrant populations (higher immigrants from other cities increase local medical burdens), and were comparatively less developed than other municipalities (Chongqing and Tianjin). Chengdu for example has high prices, high house prices, and a large population, and due to its geographic location, it does not have abundant available resources for development, all of which places a significant burden on the economy, the environment, and the population, which in turn reduces the economic efficiency and the medical input efficiency.

(3) Characteristics of cities with efficiencies above 0.5 in both stages

Twenty cities had efficiencies greater than 0.5 in both stages, with an average efficiency in the first stage of 0.8339, a standard deviation of 0.1738, and a median of 0.9350, and average efficiency in the second stage of 0.8131, a standard deviation of 0.1872, and a median of 0.8894. However, there were two main issues:

1. There were large differences between the mean and median efficiencies in the two stages, and there were outliers (maximum or minimum). With the outliers, the median should describe central trends.
2. The median efficiencies in the first stage were about 5% higher than in the second stage, and the total first stage efficiencies were slightly higher than in the second stage.

The two-stage efficiency analysis indicated that out of the 31 cities, 20 cities were performing well as they had efficiencies over 0.5 in both stages; however, the performance of the average production efficiency in the first stage was slightly higher than the average health efficiency in the second stage.

#### 4.4.2. Two stage Relative Change Rate

A relative change rate was used to assess the efficiency score change trends in the two stages; relative change rate = (second stage efficiency score − first stage efficiency score)/first stage efficiency score. A negative relative change rate indicated that the city’s second stage efficiency was lower than the first stage efficiency. Conversely, a positive relative change rate indicated that the city’s second stage efficiency was higher than the first stage efficiency. Table 4 shows the relative change rates for the 20 cities with two stage efficiencies greater than 0.5. As can be seen in Table 4 there were are eight cities with negative relative change rates, with a median relative change rate value of −0.1184. There were six cities with a positive relative change rate, with a median relative change rate value of 0.0626, which indicated that the efficiency improvements from the first stage to the second stage were less than the reduction in efficiency. Of the cities with negative relative change rates, Shanghai (−0.4472) and Hangzhou (−0.20431) had outliers. Of the cities with a positive relative change rate, the city with the largest change was Lanzhou (0.5673). As Lanzhou is a western city, this indicated that efficiency improvements are not necessarily reliant on the amount of capital. In fact, without the necessary adjustment measures, if the city were inefficient, capital accumulation could result in a waste of resources, as can be seen in the total efficiency scores in Table 5. The total efficiency scores in the western region cities of Lhasa, Urumqi, and Yinchuan were 1, while the total efficiency scores in the eastern region cities of Haikou, Hangzhou, and Jinan were very low (0.4167, 0.6063, and 0.6018), which indicated that the more developed eastern regions were not performing better than the western regions in terms of their rational use of resources. Therefore, there is an urgent need in China to optimize energy, economic, and medical input efficiencies.

**Figure 3:**
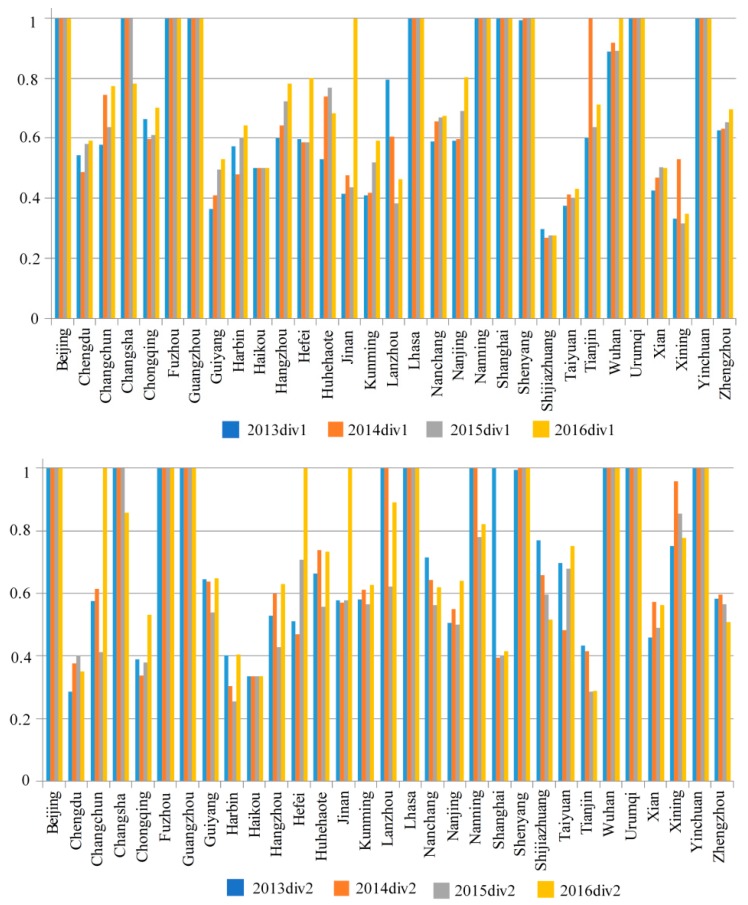
Efficiency scores in the first and second stage from 2013–2016.

Figure 3 shows that from 2013 to 2016, the cities in which the efficiencies continued to rise in the first stage were Guiyang, Hangzhou, Kunming, Nanchang, Nanjing, Xian, and Zhengzhou. However, while the other cities experienced fluctuations, the overall efficiency in the first stage in 2016 was higher than in the previous three years, indicating that in general, the production input and output efficiencies in the first stage improved.

The second stage shows that from 2013 to 2016, most cities experienced fluctuating efficiencies. Of the nine cities with efficiencies of 1 in the first stage, four saw an efficiency drop in the second stage; Nanning’s efficiency dropped to around 0.8 in 2015 and 2016, Shanghai’s efficiency dropped sharply to around 0.4 from 2014 to 2016, and Zhengzhou’s efficiency in the second stage was declining from 2015, which was opposite to its efficiency movement in the first stage. Therefore, very few cities had continual efficiency rises in the second stage with most having significant fluctuations.

Compared to the first stage, Nanchang’s overall efficiency in the second stage rose, even though there were declines from 2013 to 2015. Nanning’s overall efficiency rose in the first stage; however, there were fluctuations in the second stage, with rises in 2013 and 2014, a fall in 2015, and a rise again in 2016 to over 0.6. Shijiazhuang and Tianjin’s efficiencies in the second stage declined, with Tianjin falling to below 0.3 in 2015 and 2016.

Many cities’ overall efficiency in the second stage rose significantly in 2016; in particular, Changchun’s efficiency rose from 0.4 in 2015 to 1 in 2016, in 2015, Hefei and Jinan’s efficiency scores were less than 0.7 but rose to 1 in 2016, and Wuhan’s efficiency in the first stage only reached 1 in 2016, but was 1 in all four years in the second stage, which indicated that the health input and death rate reduction efficiencies in the second stage had improved.

### 4.5. Efficiency Scores and Rankings for Labor, Fixed Assets, Energy Consumption, and GDP from 2013 to 2016

**Figure 4:**
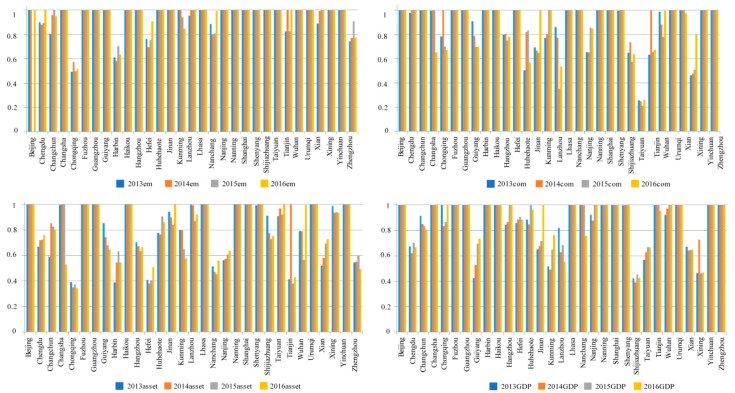
Input-output efficiency indicators for the employed population, energy consumption, fixed assets and Gross Domestic Product in the first stage from 2013–2016.

Figure 4 shows each city’s efficiency for the production input and output indicators. Of the first-stage inputs (employment, fixed assets, and energy consumption), the average employment efficiency was the highest at more than 0.9, the average fixed asset efficiency was the lowest at around 0.77, the average energy consumption efficiency was around 0.85, and the average GDP efficiency was around 0.78. Overall, employment efficiency was the highest; except for Chongqing and Harbin in which the efficiencies were lower than 0.7, most cities had efficiencies higher than 0.8, and 18 cities has efficiencies of 1. However, there were only 9 cities with overall efficiencies of 1. Generally, in most cities, the fixed asset and energy consumption input needed small adjustments.

### 4.6. Efficiency Scores and Rankings for Birth Rate and Medical Input from 2013 to 2016

**Figure 5:**
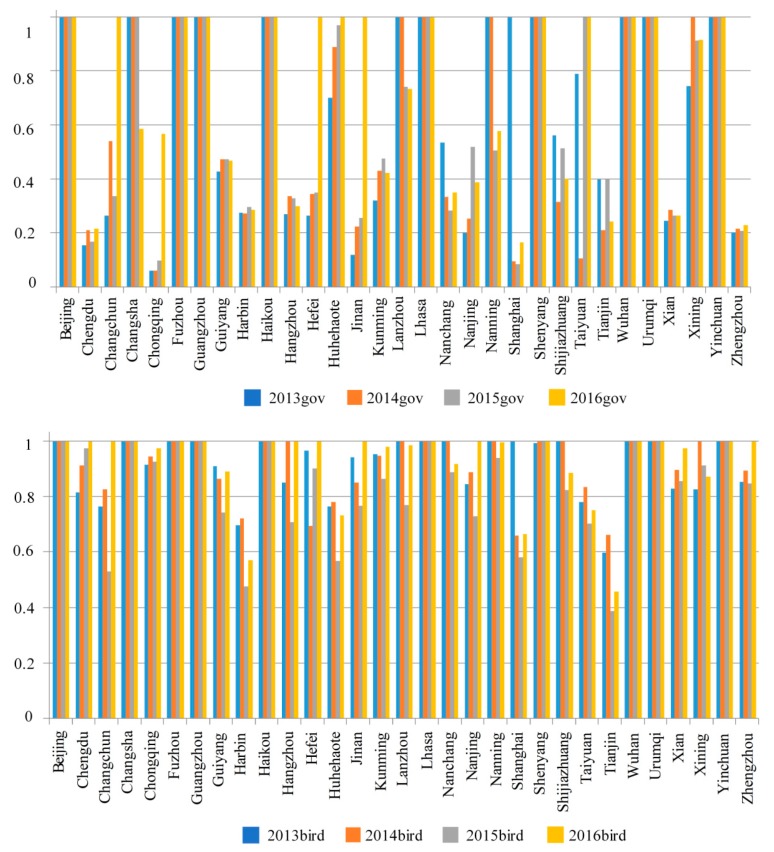
Input and output efficiencies from 2013–2016.

Table 5 and Figure 5 shows the birth rate and medical input efficiency scores in each city from 2013 to 2016. As can be seen, there were significant differences in the health input efficiencies, with most cities being very low; for example, Chengdu, Harbin, Xian, and Zhengzhou’s medical input efficiencies were less than 0.3.

### 4.7. Respiratory Disease and Death Rate Reduction Analyses in Each City

Table 6 shows the respiratory disease and death rate reduction efficiency scores in each city. The respiratory disease reduction efficiencies in Beijing, Changsha, Fuzhou, Guangzhou, Lhasa, Wuhan, Urumqi, and Yinchuan were 1, indicating high efficiency. Chengdu had the lowest respiratory disease reduction efficiency at below 0.4 in all four years, Chongqing was slightly better at below 0.6, and Hangzhou was slightly higher at close to 0.7.

The death rate reduction efficiency in Changsha in 2016 dropped slightly; however in all other years as in nine other cities, it was 1; therefore, there is no room for improvement. Although the death rate reduction efficiency was slightly higher than the respiratory disease reduction efficiency in Chengdu and Chongqing, it was still low at around 0.6. Xian had a death rate reduction efficiency of around 0.7 and Guiyang, Hangzhou, Kunming, Nanchang, Nanjing, and Shijiazhuang had efficiencies of around 0.8. Therefore, generally, the respiratory disease and death rate reduction efficiencies were high (more than 0.7); however, Chongqing and Chengdu need improvements.

## 5. Conclusions and Policy Recommendations

### 5.1. Conclusions

This paper used a DNSBM model to analyze two stages; production efficiency and health efficiency; in 31 provincial capital cities in China, and identified the areas that needed improvement as a reference for reform, health promotion, and socio-economic development. The following conclusions were made from the overall analysis:

(1) As only five cities had overall efficiencies of 1, 26 cities need improvement. The total efficiencies and the efficiencies in the two stages varied widely across the cities, which could not be explained by the eastern and western regional differences. For example, some western cities (such as Lhasa, Urumqi and Yinchuan) had higher efficiency in both stages than some eastern cities (such as Hangzhou and Haikou), which indicated that cities with better economic development do not necessarily have higher efficiency. These results clearly indicated that efficiency improvements depend on the overall development of all aspects of society such as politics, culture, education, and medical care. The analysis in this paper demonstrated that even though the eastern cities are known to have higher economic development, this does not mean that they were efficient, and pointed to the fact that China still needs to focus on economic, energy, and medical input efficiency improvements.
(2) The two stage efficiency scores in most cities had significant fluctuations, with only a few cities having a continuous rise (Guiyang, Kunming and Nanjing). This indicated again that overall efficiency is influenced by many factors. From 2013 to 2016, China experienced many major events, such as the “Internet +” action plan, industrial restructuring, supply structure reform, and “Health China 2020”. The effect of these major events and the many practical problems such as limited industrial structures, a lack of particular industries, and a lack of innovation and competitiveness, indicated the many facets of efficiency. Further, even though the central and local governments are seeking measures to improve efficiency, it is not an easy task. Therefore, it is necessary to develop effective measures to fit China’s national and regional conditions.
(3) From the analysis of the individual stage input and output efficiencies, it was evident that there needs to be improvements in government medical expenditures, with special focus needed on respiratory disease and death rate decreases, especially in Chongqing and Chengdu. Although the Chinese government’s medical input has gradually increased in recent years, the medical input efficiency had not significantly improved. Therefore, in further health care reforms, there needs to be a more rational allocation of medical resources. According to Yip and Hsiao [28], China’s primary health care is very weak and ineffective in terms of disease prevention, consultation and management, patient referrals, and medical coordination (especially in the prevention and control of non-contagious diseases). Yip and Hsiao [29] used diabetes as an example, pointing out that the hospitalization rate for diabetes complications in China was five times higher than the OECD average.

### 5.2. Policy Recommendations

Based on the results of this study, the following policy recommendations are proposed:

First, supply side structural reform is necessary. Local governments need to optimize their industrial structures rather than only pursuing GDP growth; therefore. The government should support innovation and enhance industrial competitiveness.

Second, local governments need to ensure the clean use of traditional energy as well as promoting the use of new energy. Further, when adjusting the energy structure, the government needs to focus on energy-conservation, emissions reductions, ecological protection, and pollution control to reduce the negative environmental impacts of economic development.

Third, China should improve medical investments, regulations, guidance, and supervision. Disease prevention needs to be promoted and national medical funding control policies implemented to ensure precise medical investment management and improved medical input efficiency.

## Figures and Tables

**Table 1:** Six air pollutants in China from 2013 to 2017.

Air Pollutant	Year	Average *	Range **	Reaching the Standard? ***	Proportion of Cities Reaching the Standard
SO_2_ (μg/m^3^)	2013	40	7~114	Yes	86.5%
	2014	35	2~123	Yes	88.2%
	2015	25	3~87	Yes	96.7%
	2016	22	3~88	Yes	97.0%
	2017	18	2~84	Yes	99.1%
NO_2_ (μg/m^3^)	2013	44	17~69	No	39.2%
	2014	38	14~67	Yes	62.7%
	2015	30	8~63	Yes	81.7%
	2016	30	9~61	Yes	83.1%
	2017	31	9~59	Yes	80.1%
PM_10_ (μg/m^3^)	2013	118	47~305	No	14.9%
	2014	105	35~233	No	21.7%
	2015	87	24~357	No	34.6%
	2016	82	22~436	No	41.7%
	2017	75	23~154	No	47.0%
PM_2.5_ (μg/m^3^)	2013	72	26~160	No	4.1%
	2014	62	19~130	No	11.2%
	2015	50	11~125	No	22.5%
	2016	47	12~158	No	28.1%
	2017	43	10~86	No	35.8%
O_3_ (μg/m^3^)	2013	139	72~190	Yes	77.0%
	2014	140	69~210	Yes	78.2%
	2015	134	62~203	Yes	84.0%
	2016	138	73~200	Yes	82.5%
	2017	149	78~218	Yes	67.8%
CO (mg/m^3^)	2013	2.5	1.0~5.9	Yes	85.1%
	2014	2.2	0.9~5.4	Yes	96.9%
	2015	2.1	0.4~6.6	Yes	96.7%
	2016	1.9	0.8~5.0	Yes	97.0%
	2017	1.7	0.5~5.1	Yes	98.8%

* The average for SO_2_, NO_2_, PM_10_ and PM_2.5_ refers to the annual average concentrations; the O_3_ average is the daily maximum 8 h average; and the CO average is the daily average. ** The SO_2_, NO_2_, PM_10_ and PM_2.5_ range refers to the annual average concentrations; the O_3_ range is a two-sided 90 per cent percentile for the daily maximum 8 h average; and the CO average is a two-sided 95 per cent percentile for the daily average. *** The standard here refers to the national secondary air quality standard for residential areas. The thresholds set for each air pollutant are: SO_2_ (60 μg/m^3^), NO_2_ (40 μg/m^3^), PM_10_ (70 μg/m^3^), PM_2.5_ (35 μg/m^3^), O_3_ (160 μg/m^3^) and CO (4 mg/m^3^).

**Table 2:** Annual efficiency by city from 2013 to 2016.

DMU	Overall Score	Rank	2013 (1)	2014 (1)	2015 (1)	2016 (1)
Beijing	1	1	1	1	1	1
Chengdu	0.4658	28	0.4263	0.4421	0.5063	0.4948
Changchun	0.6432	15	0.5767	0.6785	0.4996	0.8751
Changsha	0.9547	9	0.9992	0.9999	0.9998	0.8199
Chongqing	0.5254	22	0.5205	0.4758	0.4991	0.6148
Fuzhou	1	1	1	1	1	1
Guangzhou	1	1	1	1	1	1
Guiyang	0.5069	25	0.4535	0.4964	0.5168	0.5832
Harbin	0.4248	29	0.4725	0.3774	0.366	0.4901
Haikou	0.4167	30	0.4167	0.4167	0.4167	0.4167
Hangzhou	0.6063	18	0.5643	0.6218	0.5502	0.7066
Hefei	0.6474	14	0.556	0.5213	0.6475	0.8939
Huhehot	0.6699	13	0.6012	0.7384	0.6331	0.7115
Jinan	0.6018	19	0.4808	0.5184	0.5049	1
Kunming	0.5196	23	0.4696	0.4835	0.5401	0.6076
Lanzhou	0.6777	12	0.8884	0.7573	0.4961	0.6167
Lhasa	1	1	1	1	1	1
Nanchang	0.6411	16	0.6521	0.6497	0.6124	0.6502
Nanjing	0.6018	19	0.547	0.5733	0.5807	0.7211
Nanning	0.9488	10	0.9999	0.9998	0.887	0.9105
Shanghai	0.7096	11	1	0.6348	0.6214	0.6488
Shenyang	0.9985	7	0.9946	0.9996	1	1
Shijiazhuang	0.3891	31	0.4368	0.378	0.3893	0.3542
Taiyuan	0.5185	24	0.511	0.4428	0.5371	0.5819
Tianjin	0.475	27	0.4966	0.6479	0.3834	0.4247
Wuhan	0.9615	8	0.943	0.9586	0.9456	0.9995
Urumqi	1	1	1	1	1	1
Xian	0.4937	26	0.4416	0.5128	0.4966	0.5253
Xining	0.5347	21	0.4831	0.711	0.4972	0.4974
Yinchuan	1	1	1	1	1	1
Zhengzhou	0.6064	17	0.6038	0.6133	0.6062	0.602

DMU: Decision Making Units.

**Table 3:** City efficiencies for the first and second stages from 2013 to 2016.

DMU	Overall Score	Rank	Div1 (0.5)						Div2 (0.5)					
			2013	2014	2015	2016	Average	Rank	2013	2014	2015	2016	Average	Rank
Beijing	1	1	1	1	1	1	1	1	1	1	1	1	1	1
Chengdu	0.4658	28	0.543	0.487	0.582	0.591	0.5507	24	0.29	0.38	0.401	0.351	0.3534	29
Changchun	0.6432	15	0.5778	0.745	0.639	0.774	0.6839	14	0.58	0.61	0.412	1	0.6503	17
Changsha	0.9547	9	0.999	1	1	0.782	0.9451	10	1	1	1	0.858	0.9642	9
Chongqing	0.5254	22	0.6633	0.598	0.611	0.701	0.6433	19	0.39	0.34	0.379	0.531	0.4093	27
Fuzhou	1	1	1	1	1	1	1	1	1	1	1	1	1	1
Guangzhou	1	1	1	1	1	1	1	1	1	1	1	1	1	1
Guiyang	0.5069	25	0.3635	0.411	0.495	0.53	0.4499	28	0.65	0.64	0.54	0.648	0.6174	20
Harbin	0.4248	29	0.573	0.48	0.603	0.643	0.5747	22	0.4	0.3	0.254	0.403	0.3406	30
Haikou	0.4167	30	0.5	0.5	0.5	0.5	0.5	25	0.33	0.33	0.333	0.333	0.3333	31
Hangzhou	0.6063	18	0.5996	0.642	0.722	0.783	0.6867	13	0.53	0.6	0.428	0.631	0.5464	25
Hefei	0.6474	14	0.5972	0.588	0.588	0.8	0.6431	20	0.51	0.47	0.707	1	0.6714	15
Huhehot	0.6699	13	0.5298	0.74	0.768	0.684	0.6803	15	0.66	0.74	0.556	0.733	0.6723	14
Jinan	0.6018	19	0.4144	0.478	0.438	1	0.5824	21	0.58	0.57	0.577	1	0.681	13
Kunming	0.5196	23	0.4088	0.417	0.521	0.592	0.4846	26	0.58	0.61	0.566	0.628	0.5966	21
Lanzhou	0.6777	12	0.7966	0.605	0.384	0.463	0.5618	23	1	1	0.623	0.891	0.8785	11
Lhasa	1	1	1	1	1	1	1	1	1	1	1	1	1	1
Nanchang	0.6411	16	0.5888	0.657	0.67	0.676	0.648	18	0.72	0.64	0.562	0.619	0.6344	19
Nanjing	0.6018	19	0.5925	0.597	0.691	0.803	0.6708	16	0.51	0.55	0.5	0.639	0.5486	24
Nanning	0.9488	10	1	1	1	1	1	1	1	1	0.781	0.821	0.9003	10
Shanghai	0.7096	11	1	1	1	1	1	1	1	0.39	0.402	0.415	0.5528	23
Shenyang	0.9985	7	0.9941	1	1	1	0.9984	9	1	1	1	1	0.9986	8
Shijiazhuang	0.3891	31	0.2963	0.269	0.276	0.276	0.2794	31	0.77	0.66	0.596	0.516	0.6345	18
Taiyuan	0.5185	24	0.376	0.412	0.402	0.432	0.4057	29	0.7	0.48	0.679	0.751	0.6523	16
Tianjin	0.475	27	0.6023	1	0.637	0.714	0.7383	12	0.43	0.41	0.285	0.287	0.355	28
Wuhan	0.9615	8	0.8904	0.918	0.891	0.999	0.9248	11	1	1	1	1	0.9999	7
Urumqi	1	1	1	1	1	1	1	1	1	1	1	1	1	1
Xian	0.4937	26	0.4267	0.469	0.502	0.5	0.4746	27	0.46	0.57	0.489	0.564	0.5215	26
Xining	0.5347	21	0.3317	0.531	0.316	0.348	0.3815	30	0.75	0.96	0.855	0.776	0.8354	12
Yinchuan	1	1	1	1	1	1	1	1	1	1	1	1	1	1
Zhengzhou	0.6064	17	0.627	0.632	0.655	0.697	0.6527	17	0.58	0.6	0.565	0.507	0.5632	22

**Table 4:** Relative change rate in the 20 cities with two stage efficiencies greater than 0.5.

DMU	Total Efficiency Score	The First Stage Average Efficiency	The Second Stage Average Efficiency	Relative Change Rate
Beijing	1	1	1	0
Changchun	0.6432	0.6839	0.6503	−0.04913
Changsha	0.9547	0.9451	0.9642	0.02021
Fuzhou	1	1	1	0
Guangzhou	1	1	1	0
Hangzhou	0.6063	0.6867	0.5464	−0.20431
Hefei	0.6474	0.6431	0.6714	0.044006
Huhehot	0.6699	0.6803	0.6723	−0.01176
Jinan	0.6018	0.5824	0.681	0.169299
Lanzhou	0.6777	0.5618	0.8785	0.563724
Lhasa	1	1	1	0
Nanchang	0.6411	0.648	0.6344	−0.02099
Nanjing	0.6018	0.6708	0.5486	−0.18217
Nanning	0.9488	1	0.9003	−0.0997
Shanghai	0.7096	1	0.5528	−0.4472
Shenyang	0.9985	0.9984	0.9986	0.0002
Wuhan	0.9615	0.9248	0.9999	0.081207
Urumqi	1	1	1	0
Yinchuan	1	1	1	0
Zhengzhou	0.6064	0.6527	0.5632	−0.13712

**Table 5:** Medical input and birth rate efficiencies by city from 2013 to 2016.

Efficiency	City
=1	Beijing, Fuzhou, Guangzhou, Haikou, Lhasa, Shenyang, Wuhan, Urumqi, Yinchuan
<0.6	Chengdu, Chongqing, Guiyang, Harbin, Hangzhou, Kunming, Nanchang, Nanjing, Shijiazhuang, Tianjin, Xian, Zhengzhou
>0.6, <1	Huhehot, Lanzhou, Nanning, Taiyuan, Xining

The differences in health input efficiencies were greater than the differences in the birth rate efficiencies, which tended to be high in most cities, with only Harbin, Huhehot, and Tianjin having an efficiency lower than 0.8. In 2016, the medical input and birth rate efficiencies rose significantly compared to the previous three years. However, as the average medical input efficiency was low, the differences between the cities needs to be addressed.

**Table 6:** Respiratory disease and death rate reduction efficiencies in each city.

	Overall Score	Rank	2013		2014		2015		2016	
			Breath	Dead	Breath	Dead	Breath	Dead	Breath	Dead
Beijing	1	1	1	1	1	1	1	1	1	1
Chengdu	0.4658	28	0.319154	0.574365	0.379511	0.64613	0.403979	0.662443	0.308138	0.529095
Changchun	0.6432	15	0.997303	1	0.822928	0.862604	1	1	1	1
Changsha	0.9547	9	0.999742	0.99974	0.999986	0.999983	1	1	1	0.987481
Chongqing	0.5254	22	0.603698	0.6151	0.494049	0.515881	0.567756	0.561586	0.50591	0.562721
Fuzhou	1	1	1	1	1	1	1	1	1	1
Guangzhou	1	1	1	1	1	1	1	1	1	1
Guiyang	0.5069	25	0.848056	0.854939	0.866644	0.868156	0.858574	0.84627	0.856479	0.856481
Harbin	0.4248	29	0.727852	0.732364	0.494426	0.494427	0.663949	0.642656	0.919001	0.919702
Haikou	0.4167	30	0	0	0	0	0	0	0	0
Hangzhou	0.6063	18	0.684498	0.907921	0.630953	0.82618	0.648427	0.83625	0.695218	0.899324
Hefei	0.6474	14	0.650572	0.665066	0.834373	0.850786	1	1	1	1
Huhehot	0.6699	13	0.95108	0.951264	0.972262	0.972264	0.991562	0.979371	1	0.996874
Jinan	0.6018	19	0.840146	0.877419	0.880102	0.903538	1	1	1	1
Kunming	0.5196	23	0.750716	0.760086	0.746365	0.759292	0.743585	0.74358	0.750919	0.750914
Lanzhou	0.6777	12	1	1	1	1	0.847356	0.83677	0.980481	1
Lhasa	1	1	1	1	1	1	1	1	1	1
Nanchang	0.6411	16	0.801558	0.810759	0.796958	0.79695	0.811009	0.799423	0.838847	0.834847
Nanjing	0.6018	19	0.787322	0.804454	0.792394	0.810068	0.77166	0.771659	0.765302	0.76531
Nanning	0.9488	10	0.999991	1	0.999826	0.999862	1	0.981644	0.959953	0.933696
Shanghai	0.7096	11	1	1	0.835315	0.85977	0.996068	1	0.804818	0.9051
Shenyang	0.9985	7	1	1	1	1	1	1	1	1
Shijiazhuang	0.3891	31	0.864608	0.879941	0.8302	0.830193	0.831052	0.82729	0.671766	0.671762
Taiyuan	0.5185	24	0.942516	0.943528	0.819034	0.811193	0.969421	0.929005	1	1
Tianjin	0.475	27	0.880586	0.899683	0.810255	0.855752	0.878549	0.9359	0.777527	0.868989
Wuhan	0.9615	8	1	1	1	1	1	0.999984	1	1
Urumqi	1	1	1	1	1	1	1	1	1	1
Xian	0.4937	26	0.703679	0.715885	1	0.630384	0.745394	0.704918	0.733021	0.739352
Xining	0.5347	21	0.987678	1	0.936726	0.936704	0.946721	0.946691	0.879909	0.873636
Yinchuan	1	1	1	1	1	1	1	1	1	1
Zhengzhou	0.6064	17	0.955971	0.891323	0.955839	0.826401	0.929376	0.863725	0.54588	0.748732
